# The incidence of vasovagal reactions during earlobe piercing

**DOI:** 10.3389/fmed.2023.1103071

**Published:** 2023-01-20

**Authors:** Zi-ao Xie, Kai-li Zhang, Fang Han, Meng-yao Tang, Jue-wei Chen, Guang-peng Liu

**Affiliations:** Department of Plastic and Reconstructive Surgery, Shanghai Tenth People's Hospital, Tongji University School of Medicine, Shanghai, China

**Keywords:** ear piercing, vasovagal reaction, syncope, blood-injection-injury, young women

## Abstract

**Background:**

Vasovagal reactions are common amongst patients with a fear of needles receiving injections or during venipuncture, but they are rarely studied in healthy people undergoing earlobe piercing. The purpose of this prospective study was to evaluate the incidence and the features of vasovagal reactions observed during earlobe piercing.

**Methods:**

Thousand eight hundred and sixty six participants aged older than 13 years had their earlobes pierced in our department from January 2020 to January 2022. When vasovagal reactions occurred during the procedure (e.g., dizziness, pallor, diaphoresis, and faintness, etc.), they were recorded and more detailed demographic information was collected.

**Results:**

A total of 196 cases of vasovagal reactions were reported in females amongst 1,866 participants, including 58 who actually lost consciousness during earlobe piercing. The incidence of vasovagal reactions and vasovagal syncope was 10.5 and 3.11% respectively. All syncopal reactions occurred in persons younger than 30 years.

**Conclusion:**

Vasovagal syncope is often very sudden and occurs without warning. Practitioners need to be familiar with these reactions, and prevent people from an unpredictable fall and subsequent injury during ear piercing.

## 1. Introduction

Anxiety and fear related to needle injection is a commonly psychological reaction among normal people. Approximately 10% of patients in healthcare settings have a documented fear of needles which can cause subsequent avoidance and embarrassment (1). In some severe cases the fear is so intense and persistent that it warrants the diagnosis of needle phobia, a type of blood-injection-injury (BII) phobia marked by excessive fear and avoidance of injections, and/or distress due to this fear (2). Bienvenu et al. (3) reported that 1.6% of individuals in the general population are needle phobic, and that many aspects of their lives have been significantly influenced by this fear and associated avoidance, such as willingness to receive venipuncture, donate blood and job choice.

BII phobia is characterized by a variety of vasovagal reactions, including weakness, dizziness, nausea and fainting or syncope (4). A large proportion of people with needle phobia have experienced the feeling of faintness when exposed to needles and 56% of them have lost consciousness during injection or blood drawing (4, 5). Vasovagal reactions are more common among blood donors, and fainting is observed in 8% of late adolescents and 2–3% of adults who donate blood (6). Although most vasovagal reactions are usually not harmful, some people may suffer sudden syncope and subsequent traumas, such as lacerations, concussions and even bone fractures (7).

Ear piercing has been enjoying great popularity around the world for several decades, especially among adolescents and young adult women. Ear lobes are the site most frequently pierced and a needle thrust is the method most commonly employed (8). Complications related to ear piercing have been extensively reported in the literature, including local infection, bleeding, viral transmission, and keloid scarring (9, 10). However, few studies thus far have focused on the occurrence of vasovagal reactions during the process of ear piercing. In our clinical practice, a variety of adverse responses, such as nausea, dizziness, and vasovagal syncope, are often observed in individuals upon exposure to earlobe piercing, a specific type of needle stimulus.

In this study, we investigated the incidence and features of vasovagal reactions in 1,866 patients who received ear piercings in our department. More and more people like to have their ears pierced in hospital because they believe that the procedure performed by medical staff can reduce the risks of bacterial infections and disease transmission. Thus, our hospital-based setting presents a unique opportunity to evaluate the occurrence of adverse reactions among people during earlobe piercing and study demographic and psychological characteristics associated with these vasovagal reactions.

## 2. Materials and methods

### 2.1. Participants

This prospective study was approved by the ethical review board of Shanghai Tenth People's Hospital. A total of 1,890 individuals underwent earlobe piercing in our department between January 2020 and January 2022. Except for 24 children under 13 years, 1,866 participants were enrolled in this study. Among them, 51 (2.73%) are males and 1,815 (97.27%) are females, with the mean age of 27.26 years (range 13 to 75 years). The earlobe thrust was performed using a 20-G sterile needle in an anterior to posterior direction without local anesthesia. The practitioners consisted of five plastic surgeons and one experienced nurse.

### 2.2. Data collection

When vasovagal reactions occurred during piercing, the practitioner stopped immediately the procedure, recorded these reactions and provided proper service to ensure participant health and safety, such as letting him or her lie down and/or providing some candy. The vasovagal reactions were divided into two categories: responses without syncope (e.g., dizziness, pallor, diaphoresis, nausea, emesis, etc.), and actually loss of consciousness. After recovery, a follow-up interview with the individual was completed to collect more detailed information of interest (Table 1). In this subjects with syncope the prodromes were not well-collected, which does not affect the results.

**Table 1:** Data collection.

**Recorded data**
Operation date
Age
Sex
History of syncopal reaction
After-puncture symptoms
Dizziness
Pallor
Amaurosis
Nausea
Diaphoresis
Chest discomfort
Chills
Loss consciousness

Detailed information of participants with vasovagal reactions.

## 3. Results

One hundred and ninety six female individuals with vasovagal reactions (reactors) were recorded of 1,866 participants in this study, including 58 cases who experienced vasovagal syncope during earlobe piercing. The occurrence of vasovagal reactions was 10.5% (196/1,866) and the incidence of syncopal reaction was 3.11% (58/1,866). The average age of the reactors without syncope (*n* = 138) was 20.58 years (range 15 to 33 years). Dizziness (31%), pallor (30%), and nausea (14%) were the three most common responses recorded (Table 2). Adverse reactions occurred in 8 cases after the first-earlobe piercing (5.8%), and in 130 cases after the second puncture (94.2%).

**Table 2:** Clinical findings.

**Clinical findings**	***n***	**Proportion**
Vasovagal reactions	138	
Dizziness	43	31.16%
Pallor	42	30.44%
Nausea	20	14.49%
Diaphoresis	11	7.97%
Chest discomfort	11	7.97%
Amaurosis	6	4.35%
Chills	5	3.62%
Vasovagal syncope	58	

Number of people and incidence of symptoms.

**Figure 1:**
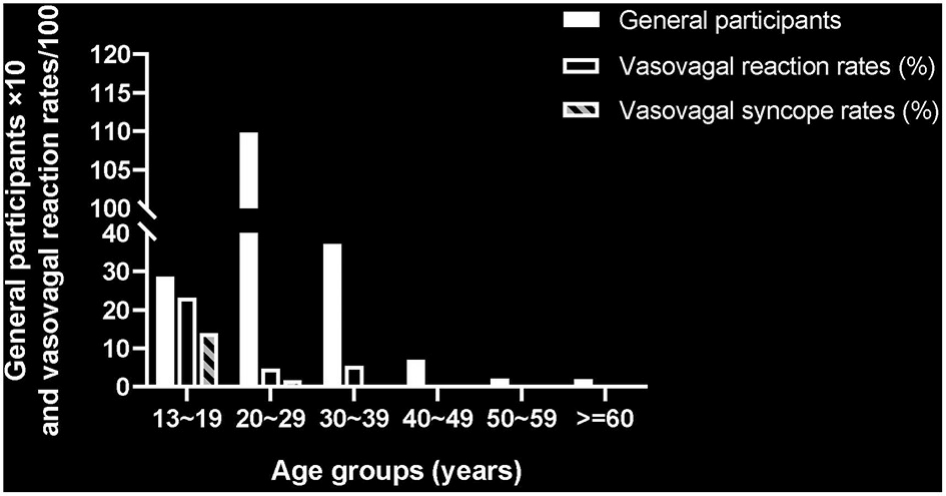
Comparison of participant's numbers and the vasovagal reaction rates.

Figure 1 demonstrates that the 58 cases with syncopal reactions were all younger than 30 years, including 40 adolescents aged <20 years and 18 adults between 20 and 29 years. The mean age of these syncopal cases was 18.71 years (range 15 to 26 years), significantly lower than the average age of the general participants (27.26 years). The syncopal fainting is sometimes preceded by some prodromes, such as dizziness and nausea. All syncope occurred during or immediately after the second-earlobe piercing. Except for one 18-year girl who claimed to have experienced syncope during previous venipuncture, no others reported a history of vasovagal syncope. The duration of syncope was <1 min in 45 cases, and between 1 and 2 min in 12 cases. A 17-year girl experienced the longest faintness of approximate 150 s. They were relieved after resting in bed for a few minutes and no syncope-associated injuries were reported.

## 4. Discussion

To our knowledge, this is the first study to report the incidence of vasovagal reactions among people during earlobe piercing and to describe the demographic characteristics associated with these reactions. Our results showed that 196 of 1,866 cases experienced vasovagal reactions, including 58 young women who lost consciousness during the procedure. The incidence of vasovagal syncope was 3.11%, similar to that among blood donors (2.6%) (6).

Most participants with adverse reactions in our study received ear-piercing voluntarily, and did not show higher anxiety or excessive fear before the procedure. Therefore, we speculate these vasovagal responses were not elicited by needle phobia, but by the stimulus of earlobe puncture, an invasive medical procedure. Fainting in the presence of BII stimuli is often observed among women of younger age (1, 2), consistent with the main findings of our present study. The onset of vasovagal syncope is quite sudden and occurs without warning. If practitioners are familiar with these reactions and able to recognize them quickly, an unpredictable fall and subsequent traumatic injury may be prevented.

There are some limitations to this study. First, it was conducted in only one hospital from 1,866 participants. A larger sample size from more units will provide more comprehensive conclusion and evaluation. Second, we did not use a psychometrical questionnaire to assess the mental features of these samples, such as anxiety, nausea, pain, etc. In addition to younger age and female gender, other more precise factors predicting vasovagal reactions in ear piercing warrant investigation. Third, since 97% of the patients were female, our reported prevalence of vasovagal reactions and vasovagal syncope is only valid for the female sex and not for the general population.

## Data availability statement

The raw data supporting the conclusions of this article will be made available by the authors, without undue reservation.

## Ethics statement

The studies involving human participants were reviewed and approved by the Ethical Review Board of Shanghai Tenth People's Hospital (No. 20200012). Written informed consent to participate in this study was provided by the participants' legal guardian/next of kin.

## Author contributions

Z-aX wrote the first draft of the manuscript and performed the statistical analysis. G-pL and Z-aX contributed to conception and design of the study. G-pL wrote sections of the manuscript. K-lZ, FH, M-yT, and J-wC organized the database. All authors contributed to manuscript revision, read, and approved the submitted version.
